# A full-body motion capture gait dataset of 138 able-bodied adults across the life span and 50 stroke survivors

**DOI:** 10.1038/s41597-023-02767-y

**Published:** 2023-12-01

**Authors:** Tamaya Van Criekinge, Wim Saeys, Steven Truijen, Luc Vereeck, Lizeth H. Sloot, Ann Hallemans

**Affiliations:** 1 Department of Rehabilitation Sciences, KU Leuven Campus Bruges, Bruges, Belgium; 2 Research group MOVANT, Department of Rehabilitation Sciences & Physiotherapy, University of Antwerp, Wilrijk, Belgium; 3 Institut für Technische Informatik (ZITI), Heidelberg University, Heidelberg, Germany; 4 Translational and Clinical Research Institute (TCRI), Newcastle University, Newcastle, UK

**Keywords:** Stroke, Geriatrics

## Abstract

This reference dataset contains biomechanical data of 138 able-bodied adults (21–86 years) and 50 stroke survivors walking bare-footed at their preferred speed. It is unique due to its size, and population, including adults across the life-span and over 70 years, as well as stroke survivors. Full-body kinematics (PiG-model), kinetics and muscle activity of 14 back and lower limbs muscles was collected with a Vicon motion capture system, ground-embedded force plates, and a synchronized surface EMG system. The data is reliable to compare within and between groups as the same methodology and infrastructure were used to gather all data. Both source files (*C3D*) and post-processed ready-to-use stride-normalized kinematics, kinetics and EMG data (*MAT-*file, Excel file) are available, allowing high flexibility and accessibility of analysis for both researchers and clinicians. These records are valuable to examine ageing, typical and hemiplegic gait, while also offering a wide range of reference data which can be utilized for age-matched controls during normal walking.

## Background & Summary

Since walking ability is for many patients a primary goal during rehabilitation, a thorough understanding of normal and pathological gait processes is required to distinguish healthy ageing from pathology. Instrumented gait analysis is the best method to examine walking patterns and determine gait deficits and treatment strategies^1^, but it is time-consuming and requires extensive preparations and data processing. Thus, large motion capture datasets are scarce. To enhance our knowledge of ageing processes and gait-related research questions, we need reliable datasets from healthy adults across the lifespan that include motion, forces and muscle activity data. When comparing normal and pathological gait, we need to consider the natural aging process and sex, as patient groups are usually older and sex differences in gait kinematics persist across different speeds^2^. Hence, sex and age-matched control data is essential for interpreting gait deficits.

Nevertheless, available datasets on able-bodied adults are rather small, include solely lower limb data, while focusing on either kinematics, kinetics or muscle activity instead of combining measurements^3–9^. The limited full-body reference data available are inadequate to aid us in understanding the ageing process as they do not include adults over the age of 72 years^9–12^, yet gait changes still occur after this age^13–17^. On the other hand, only a few gait datasets have been published in people with neurological diseases^9,18^, to our knowledge, no full-body motion capture datasets incorporating kinetics and EMG are currently available of people post-stroke.

This reference dataset is unique as it contains kinematic, kinetic and muscle activity measurements during bare-footed walking at preferred speed of 138 able-bodied adults across the life span (21–86 years) as well as over the age of 70 years, as well as biomechanical and clinical data of 50 stroke survivors. The same methodology and infrastructure was used to gather all data from both datasets, making this a reliable dataset to compare within and between groups. In addition, to allow high flexibility and accessibility of data usage for both researchers and clinicians, both source files (*C3D*) and post-processed ready-to-use stride-normalized data in a MATLAB structure (*MAT-file*) are available.

This dataset was already instrumental in showing that ageing results in a decline in peak muscle amplitude^17^, center of mass push-off power^16^, joint range of motion^19^ and mediolateral margins of stability^20^, while not affecting the dynamic balance margins of gait^21^. In addition, the stroke dataset has already supported the identification of trunk impairments during walking after stroke^22^. Although this dataset has been used in the previous studies to describe age-related decline during walking, there are still many open questions and challenges that require further investigation and exploration. Additionally, building large data sets with opto-electronic devices takes time, sharing this dataset allows for collaborative science resulting in big data projects. We do acknowledge that this dataset is does not allow for speed-analysis as only self-selected speeds are included. Nevertheless, these records are exceptionally valuable to examine ageing processes during walking, to answer fundamental research questions concerning typical and hemiplegic gait, while also offering a wide range of reference data which can be utilized for age-matched controls during normal walking. Forthcoming insights from this dataset might result in the optimization of gait and balance training for stroke survivors and ageing adults, which in turn could lead to greater participation in society and independence.

## Methods

### Participants

In total, 138 able-bodied individuals (21–86 years) and 50 stroke survivors (19–85 years) were included in the datasets, as seen in Table 1.

**Table 1:** Characteristics of the dataset.

	Able-bodied adults (n = 138)		Stroke survivors (n = 50)	
	Mean (SD)	Min-Max	Mean (SD)	Min-Max
Age (y)	51 (20)	21–86	64 (14)	19–85
Gender (m/f)	65/73		34/16	
Body mass (kg)	74 (15)	48–157	72 (14)	50–106
Height (mm)	1684 (103)	1420–1920	1705 (80)	1500–1890
Body Mass Index	26 (4)	18–47	25 (4)	18–35
Leg length (mm)	899 (61)	660–1070	882 (46)	790–970
Time post stroke (days)			53 (19)	14–139
Type of stroke (I/H)			39/11	
Lesion location (L/R)			17/33	
Functional Ambulation Category			3 (1)	2–5
Trunk Impairment Scale			14 (3)	7–20
Tinetti POMA			19 (6)	6–28

ID: identification number, Min: minimum, Max: Maximum, y: years, M: male, F: female, kg: kilograms mm: millimetre, L: left, R: right, I: ischeamic, H: hemorrhagic, Tinetti POMA: Tinetti Performance Oriented Mobility Assessment.

Able-bodied participants were excluded from the study if they had self-reported visual impairments, an antalgic gait pattern, abnormal mobility in the lower limbs or any known neurological or orthopaedic disorder that could influence motor performance and balance. No medical records were obtained from these volunteers. People post-stroke were included in this dataset if they met the following criteria: 1) haemorrhagic or ischaemic stroke diagnosis, confirmed based on CT or MRI imaging; 2) no known history of previous stroke; 3) stroke onset within five months; and 4) age between 18 and 85 years. Information concerning stroke diagnosis, medical history, and stroke onset was acquired via medical records.

Since stroke survivors were also enrolled in a randomized controlled trial, there were several exclusion criteria: (1) they ≥ 20 on the TIS which indicates normal truncal function^23^; (2) they had a Functional Ambulation Score ≤ 2 as participants needed to be able to ambulate without continuous physical support to ensure that gait analysis can be executed safely; (3) they were not able to sit independently, without support and supervision, for 30 seconds on a stable surface; (4) they suffered from other neurological and orthopaedic disorders that could influence motor performance and balance; and (5) they were not able to understand instructions.

Informed consent was obtained prior to data collection. Ethical approval of the study according to the Declaration of Helsinki was obtained from the local ethics review committee (The following reference numbers were used during the application: 15/42/433, 151203ACADE and B300201316328). Trial registration on ClinicalTrials.gov (ID: NCT02708888)

### Instrumentation and materials

Participants received a full-body instrumented gait analysis performed in the Multidisciplinary Motor Centre Antwerp (M2OCEAN) movement analysis laboratory. The study used a three-dimensional passive motion capture system (8 Vicon T10 cameras, ©Vicon Motion Systems Ltd., Oxford, UK, 100 frames per second, resolution 1 Megapixel 1120 × 896), 4 ground embedded force plates (3 AMTI type OR 6–7 force plates, 1000 frames per second, 46 × 50 × 8 cm, ©Advanced Mechanical Technology, Inc., Watertown, USA; and 1 AccuGait® force plate, 1000 frames per second, 50 × 50 × 4 cm, ©Advanced Mechanical Technology, Inc., Watertown USA) and a synchronized 16-channel telemetric wireless surface EMG system (Zerowire, ©Cometa, Barregio, Italy) for data collection. Additional material consisted of reflective markers (14 mm, ©B&L Engineering, California, USA) to measure kinematics and spatiotemporal parameters, and disposable gel electrodes (Covidien Kendall^TM^, 30 mm × 24 mm) to measure EMG activity. Information concerning the calibration and reliability of the system can be found in the technical validation paragraph.

### Data collection protocol

Before arrival of the participant, systems were calibrated according to the instructions provided by the manufacturer. When the participant arrived, they received more detailed information about the upcoming protocol. For the able-bodied participant, informed consent was signed at the same day of testing, while informed consents were already collected in the stroke survivors prior to their lab visit. After any questions participants had were answered, participants were prepared in the following standardized manner. They were asked to change into tight-fitting shorts and short sleeve t-shirts without shoes, to ensure all markers were placed on the skin and not covered by clothes. Nor did they wear a safety harness or were otherwise restrained during data collection.

First, the following anthropometric measurements were taken: body mass, height, and leg length (measured as the longitudinal distance between spina iliaca anterior superior and malleoli medialis in supine). Additionally we collected descriptive characteristics of the stroke survivors including time since the stroke, type of stroke, lesion location, Functional Ambulation Categories (FAC)^24^, Trunk Impairments Scale (TIS)^25^ and Tinetti Performance Oriented Mobility Assessment^26^. These performance measures were collected on a different day to prevent fatigue in the participants post-stroke during walking. Detailed information per individual can be found in the Supplementary Table 1.

Second, fourteen EMG electrode placement locations were palpated according to the SENIAM guidelines^27^ and confirmed by selective muscle contractions. If participants post-stroke were not able to activate selective muscle, electrode placement was solely based on SENIAM guidelines. To ensure good electrode-skin contact and to minimize the risk of artefacts in the EMG signals, the skin at the placement location was properly prepared by shaving and degreasing. The following muscles were included on both the left and right side: m. rectus femoris (LRF, RRF), m. vastus lateralis (LVL, RVL), m. biceps femoris (LBF, RBF), m. semitendinosus (LST, RST), m. tibialis anterior (LTA, RTA), m. gastrocnemius (LGAS, RGAS) and m. erector spinae (LERS, RERS). Only 14 channels were used so that the additional two EMG channels were available when practical problems arose and channels could be switched (NOTE: all EMG channels in the source datafiles are labelled based on muscle abbreviations).

**Fig. 1:**
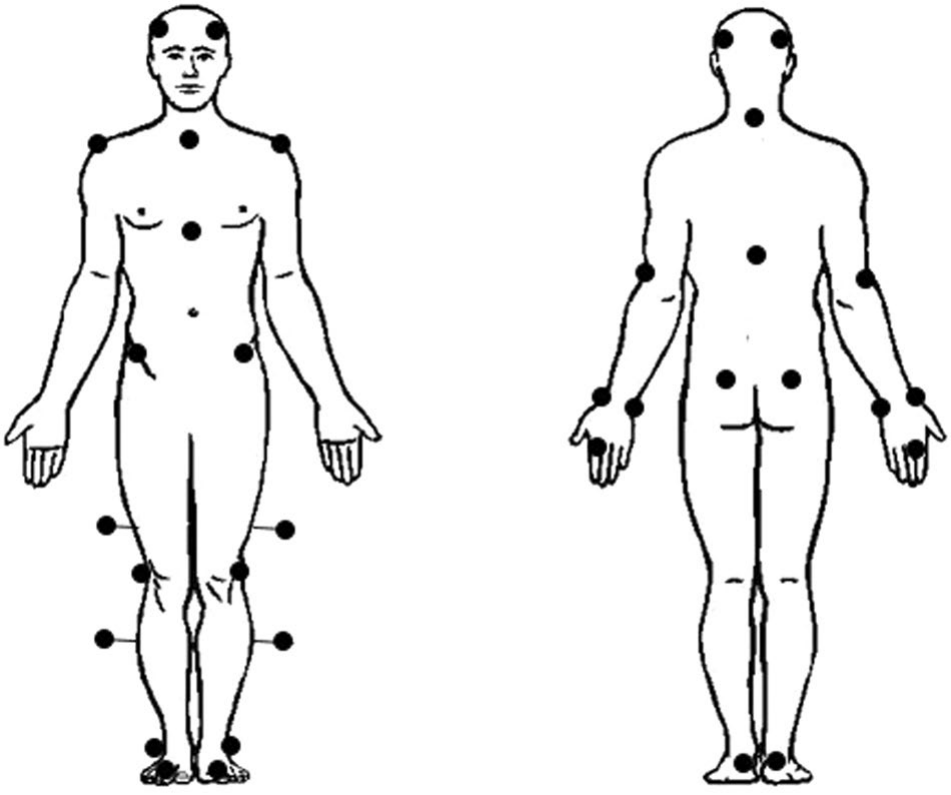
Plug-In Gait model.

Third, reflective markers were attached to bony anatomical landmarks according to the standard Plug-In Gait full body model^28,29^, as seen in Fig. 1. Bony landmarks were located by manual palpation by the same investigator who underwent extensive training. Reflective markers were firmly affixed to the skin twice (double-sided tape and skin-sensitive tape).

At last, EMG sensors were attached to the surface electrodes and affixed to the skin with double-sided tape and skin-sensitive tape. The signal to noise ratio was checked in the Vicon software by asking participants to maximally contract each muscle (if possible) to ensure clear EMG-recordings.

When subject preparation was completed, a static calibration was performed with the knee alignment device which aids in defining the flexion axis of the knee^30^. Participants were asked to stand still in the middle of one force platform with arms fully extended to the sides and thumbs facing down (i.e. base pose defined by the Vicon user guide). The static trial was checked for full marker visibility and repeated if needed.

Subsequently, participants walked over a 12-meter walkway at a self-selected speed. Participants walked without walking aids or orthoses. Participants were instructed “to walk as usual, not to mind the markers on their body, to look forward and not to interact with anyone during the walking trials”. No information was provided concerning the purpose of the ground-embedded force plates to avoid any targetting. To maximize safety for the stroke survivors, a skilled physiotherapist walked next to the participant on the non-hemiplegic side providing no assistance. This did not affect the visibility of the markers.

The aim was to record a minimum of six walking trials during steady-state walking phase without gait initiation and termination, and with three ‘correct’ heel strikes on the force plates from the right and three from the left stride without contact of the other foot on the force plate. However, for stroke survivors and some older participants, these requirements were sometimes deemed too fatiguing, resulting in a lower number of recorded trials in two subjects and the need for physical assistance (i.e., walking while holding hand of physiotherapist) in three subjects. A detailed description per participant of how many clean strikes were retrieved and the need for assistance can be found in the data records paragraph.

### Data processing

Reflective markers were tracked and labelled with the Vicon Nexus software (over the years: software packages 1.8.5 to 2.10.1). Gap filling was performed manually using pattern or spline fills based on the most accurate trajectories and limited to a maximum of 20 consecutive frames. Automatic pipelines were not used for gap filling to control marker trajectory quality. Based on the ankle and heel marker trajectories and force plate recordings (force threshold 10 N), events of foot strike and toe-off were determined in Vicon Nexus. All events were visually checked before further processing. Subsequently, the Plug-In Gait Dynamic pipeline was run which includes a Woltring filter based on a fifth-order interpolating function with a MSE smoothing value of 10, and incorporates the morphologic characteristics of the participant (e.g, body mass, height, leg length, and joint widths) in the final dynamic model when calculating kinematics (i.e., 3D full-body joint angles, and 3D center of mass) and kinetics (i.e., 3D joint forces, 3D joint moments and 3D joint power). Detailed information about this pipeline can also be found in the online documentation provided by the manufacturer^28,29^. At last, processed data was exported to *C3D* files. The final selection of these files with good strides are shared with this dataset.

To create the Matlab-postprocessed data file, further postprocessing was performed in Matlab (version R2021a, Mathworks, Natick, MA, USA). First, the identified gait events in Vicon Nexus were checked. In Matlab, toe-off was defined as the minimum in the ankle angle in a time window of 30 samples around the toe-off values identified in Vicon Nexus. Initial contacts were compared to the onset of vertical ground reaction force for those strides that had a good force plate landing. Initial contacts and toe-off events were visually checked. Second, all marker, kinematic and kinetic data were filtered using a 4^th^-order reversed Butterworth filter with a low-pass filter frequency of 10 Hz. Rectified EMG data was bandpass filtered with a 2^nd^-order reversed Butterworth filtered passing frequencies between 10 and 300 Hz, and then smoothed with a 50 ms moving average filter to generate the linear envelope^17^. EMG data were normalized to the maximum found value over the available strides per muscle^31^. Third, kinematic and EMG data of all strides was time-normalized to a stride with 1000 datapoints, but kinetic data were only time-normalized for the strides with a good force plate landing for the able-bodied dataset. These good strides were identified by visually checking the C3D files from Vicon Nexus in Visual3D. For the stroke dataset, such manual checks were not performed and data was time-normalized for all available kinematic strides. Note that the available strides of a specific participant were collected from both within the same trial as well as between different walking trials. However, the matlab structure provides the corresponding trial number for each stride, if this is relevant for a project. Fourth, the walking direction was determined from the heel data, and corrections were made in the anterior-posterior direction and medio-lateral direction. Gait events and other relevant data were added to the created MAT structure (for a more complete description, see Data Records).

## Data Records

Both the source and data files (*50_StrokePiG* and *138_HealthyPiG*) as well as the post-processed structures (*MAT_normalizedData_AbleBodiedAdults_v06-03-23* and MAT *normalizedData_PostStrokeAduls_v27-02-23, with a description file MATdatafiles_description_v1.3_LST*) and postprocessed summary files (MAT_normalizedData_AbleBodiedAdults_v06-03-23 and MAT_normalizedData_PostStrokeAdults_v27-02-23) both containing stride-normalized data are available at Figshare^32^. Figures showing the left and right stride data per participant for most commonly used variables (eg sagittal joint angles, moments and powers, center-of-mass, ground reaction forces, EMG data) are available together with example Matlab code (ExampleCode_LoadStruct_PlotTimeNormVar_v1) to make similar plots on Figshare. Metadata of the subjects are provided as Supplementary material in Supplementary Tables 1, 2, while a detailed description of good force plate landings per participant is provided in Supplementary Tables 3, 4. Comfortable walking speed per participants Supplementary Table 5.

The source files are stored in the *C3D* file format with no additional data processing performed (except for the standard dynamic PiG pipeline as explained above). This is the most used file format by all motion capture systems and biomechanics software and guarantees easy importing into other software for further processing.

The *C3D* file contains anthropometric measurements (e.g., body height, body mass, leg length, …), 3D kinematics (i.e., 3D marker trajectories, 3D full-body joint angles, and 3D center of mass), kinetics (i.e., 3D ground reaction forces, 3D joint forces, 3D joint moments and 1D joint power), and unfiltered EMG. All calculated are based on the Full body Plug-in Gait model and standard processing pipelines, further information is provided in the online documentation of the manufacturere manufacturer^28,29^. The *C3D* data records are provided in the folders “50_StrokePiG” and “138_HealthyPiG” containing a static (standing anatomical-calibration trial) and at least 3 dynamic (walking) trials. The dynamic *C3D* files are labelled according to the trial number: SUBJ (No) for able-bodied adults and BWA_No for stroke survivors. The subfolders are coded with sequential numbering beginning with either the letters SUBJ for able-bodied adults and TVC (abbreviation name corresponding author) for stroke survivors. The static trial is numbered as trial SUBJ (0) for all able-bodied adults and as [cal No] for all stroke survivors. Two participants have only two dynamic *C3D* files (TVC42 and TVC56) due to marker loss or excessive fatigue during walking.

To facilitate the use of the datasets in this paper, a post-processed Matlab structure (*MAT* files) is also available that contains stride-normalized data, anthropometric data, and gait events, for able-bodied participants (“*MAT_normalizedData_AbleBodiedAdults_v06-03-23*”) and post-stroke participants (“*MAT normalizedData_PostStrokeAduls_v27-02-23*”). This data includes kinematic data (i.e., 3D full-body marker data, 3D full-body joint angles, and 3D center of mass), and EMG data (i.e., both not and normalized traces to the maximum value across all strides of a subject of the ERS, RF, VL, BF, ST, TA and GAS for both legs) for all available strides, as well as kinetic data normalized to body mass (i.e., ground reaction forces, joint moments and joint powers) for those strides with clean strikes on the force plate. Clean strikes are foot contacts with a single foot on one or two force plates - allowing cross-plate strikes. In these files, selected variables were checked for outliers, and strides with outliers were set to NaN, similar to the strides with missing data such as EMG. These manual corrections were made when warranted by the authors (LHS and TVC). However, kinetic analysis in the stroke population was no priority during data collection, due to the limited amount of adequate force data, good foot landing and thus kinetic data quality and outliers were not assessed in the *MAT-files*. A description of the *MAT-file*s and the checked variables is available as a separate*.xls file* in the Figshare repository^32^ as “*MATdatafiles_description_v1.3_LST*”. A summarized version of these data structures are provided, containing the time-normalized average per participant per variable (MAT_normalizedData_AbleBodiedAdults_v06-03-23 and MAT_normalizedData_PostStrokeAdults_v27-02-23). A visual presentation (*.png*) of the selected variables in the *MAT-files* can be found in the in the Figshare repository^32^ as “*TimeNormalizedFigs_AbleBodied_v06-03-23*” and “*TimeNormalizedFigs_Stroke_v27-02-23*”, for able-bodied and stroke participants respectively, together with example Matlab code to make these plots (ExampleCode_LoadStruct_PlotTimeNormVar_v1). A README.txt file, accessible in the FigShare repository, is provided for enhanced readability and clarification of the legends associated with the graphs. The amount of strides with good kinematic and kinetic data in the post-processed MAT files are described in the*.xls file* “*NrStrides*”. The able-bodied adults had on average six good strides (taken over left and right side) for kinematic data and two for kinetic data, while the post-stroke participants had on average eight good strides for kinematic data and 1.5 good strides for kinetic data on both the paretic and non-paretic side.

Not all participants had a fully complete dataset. One hundred eleven able-bodied adults and 47 stroke survivors have accompanying analogue data recordings (i.e., EMG and force plate data). EMG data is missing for for 27 able-bodied adults (SUBJ23, 48, 54, 72, 85, and SUBJ118-138) and three stroke survivors (TVC11, TVC55 and TVC57). All EMG channels in the source datafiles are labelled based on muscle abbrevations. In addition, three subjects post-stroke (TVC48, TVC51 and TVC54) needed minimal physical support of a physiotherapist during walking by holding the non-hemiplegic hand.

## Technical Validation

### Reliability and accuracy of movement registration

Instrumented gait analysis is the current accepted gold standard for movement registration and has a strong to very strong test-retest and inter-tester reliability in all three anatomical planes^33–36^. To determine the accuracy and precision of the 3D marker coordinates obtained from the Vicon ® T10 system, the following technical validation protocol was performed. A dummy with five markers (14 mm in diameter), positioned in a pre-defined configuration of 90°, was placed within the measurement volume at four different locations: in the beginning of the walkway, in the middle of the walkway oriented according to the global reference frame, in the middle of the walkway rotated over 90° around the z-axis and at the end of the walkway. Afterwards, the dummy was moved through the measurement volume by an experimenter, with the markers oriented in the sagittal (sag), frontal (front) or transversal (trans) plane or moving in 3D through the entire measurement volume (dynamic conditions). r each condition, five recordings of each 2.00 s in duration were taken. Based on the raw (x,y,z) – coordinates of the five markers, the distances between each pair of markers were calculated according to formula (1-2) as well athe angle formed by the two arms of the dummy according to formula (3).

1 $${\bar{X}}_{1}=\frac{{\sum }_{i=1}^{n}{x}_{1i}}{n}\quad {\rm{and}}\;{\rm{similarly}}\;{\rm{for}}\;{\rm{the}}\;{\rm{y}}\;{\rm{and}}\;{\rm{z}}\;{\rm{coordinates}}$$

2 $${D}_{12}=\sqrt{{({\bar{X}}_{2}-{\bar{X}}_{1})}^{2}+{(\bar{Y}{}_{2}-\bar{Y}{}_{1})}^{2}+{({\bar{Z}}_{2}-{\bar{Z}}_{1})}^{2}}$$

3 $$\alpha {=\cos }^{-1}\left(\frac{({\bar{X}}_{2}-{\bar{X}}_{1})\cdot ({\bar{X}}_{5}-{\bar{X}}_{1})+({\bar{Y}}_{2}-{\bar{Y}}_{1})\cdot ({\bar{Y}}_{5}-{\bar{Y}}_{1})+({\bar{Z}}_{2}-{\bar{Z}}_{1})\cdot ({\bar{Z}}_{5}-{\bar{Z}}_{1})}{\sqrt{{({\bar{X}}_{2}-{\bar{X}}_{1})}^{2}+{({\bar{Y}}_{2}-{\bar{Y}}_{1})}^{2}+{({\bar{Z}}_{2}-{\bar{Z}}_{1})}^{2}}\times \sqrt{{({\bar{X}}_{5}-{\bar{X}}_{1})}^{2}+{({\bar{Y}}_{5}-{\bar{Y}}_{1})}^{2}+{({\bar{Z}}_{5}-{\bar{Z}}_{1})}^{2}}}\right)$$

Where n = number of frames per trial, i.e. 2000; (x_1i_, y_1i_, z_1i_) refer to the 3D coordinates of marker 1 in frame i; (x_2i_, y_2i_, z_2i_) refer to the 3D coordinates of marker 2 in frame i, etc.; D_12_ is the calculated distance between marker 1 and 2. Distances between other marker pairs are calculated according to a similar formula.

Accuracy of the Vicon ®T10 3D camera system was determined as the mean absolute error between the observed and true values for the distance and angular measures. True values are the values programmed by the manufacturer in the Vicon® software. Precision was defined as the mean within-trial standard deviations of the distance and angular measures. The Vicon system has an accuracy of a system error lower than 2.0 mm^37^. The angular measurement accuracy of the movement laboratory was 0.95 mm (SD 0.85 mm) and 0.138° (SD 0.07°) in static conditions, while the mean absolute error was 1.81 mm (SD 2.88 mm) and of 0.12° (SD 0.04) in dynamic conditions. Precision of the distance and angular measurements was 0.05 mm (SD 0.02 mm) and 0.02° (SD 0.003°) in static conditions, and 0.54 mm (SD 0.18 mm) and 0.27° (SD 0.20°) in dynamic conditions.

Moreover, the Plug-In gait marker model used in this study has a good intra-protocol repeatability and has been commonly used to perform full-body gait analysis. Yet variability due to differences in marker placement have shown to be the major contributor to overall variance in movement registration^38,39^, requiring knowledge and standardisation of the anatomical landmarks (i.e. placement of markers in similar body positions and standardized palpation methods) is necessary to reduce errors and limited variability in marker placement. For that reason, only the primary researcher (TVC) was in charge of marker placement in the dataset presented to optimize inter- and intra-reliability of placement. Markers were affixed with double-sided tape and additional sensitive skin tape to assure consistent placement throughout the trials. There were only a limited amount of cases were a marker fell off because we used double tape fixation. When a marker print was left on the skin we affixed it immediately at the same position. Only once we redid the static calibration as we were unable to determine the original location of the marker. In this case, only the data of the final calibration is used and included in the data records. The authors affirms reliability of marker placement through the use of a validated Vicon marker model^28,29^, comprehensive training and experience of the primary researcher, and alignment of normative data with established norms^9^.

Data processing of marker trajectories can introduce data errors when not performed and checked correctly. However, during data collection and analysis we took the following precautions to minimize this risk: 1) by visually and manually checking the data in every step of the data analysis process; 2) not using automated processes in the labelling and gap-filling steps to tightly control the accuracy of marker trajectories; 3) allowing only one experienced researcher (TVC) to perform the post-processing in Vicon Nexus; and 4) using validated and reliable software for the calculation of selected variables. More specifically, the labelling of reflective markers was for every trial performed by the same researcher, without the use of automated labelling procedures. Gap filling (i.e., a spline fill with a 20 consecutive frame limit) was performed manually and visually checked for errors and performed from the selected start to end of each cropped trial (i.e., steady-state walking phase) by the same researcher without using automated pipelines. Gait events were determined based on ankle and heel marker trajectories and force plate recordings (force threshold 10 N). All events were visually checked for accuracy and 3D trajectories were fully reconstructed without any gaps before proceeding to further processing of the static and dynamic trials. Subsequently, the standard and widely used Plug-In Gait Dynamic pipeline of the Vicon Nexus Software (versions 1.8.5 to 2.10.1) was run^28,29^, which has shown to have a high reliability (ICC > 0.76–90)^40,41^.

During data collection, the aim was to collect 3 good force plate recordings of the left foot and 3 of the right, yet this was always seen as a minimum and additional recordings were performed when the participants were capable. This usually resulted in plenty of trials that could be analysed when gaps were over the 20-frame limit and marker trajectories were not visible for the entire trial. Therefore, it is ensured that reliable and accurate kinematic trials are reported in this dataset. However, kinetic analysis was no priority during data collection and clean strikes are therefore not guaranteed in the *C3d files* for all participants and should be checked by the user of these data files. The kinetic data in the *MAT-file* for able-bodied adults, was checked for quality and outliers, and manual corrections have been made, but not for the stroke dataset.

### EMG data

Surface EMG is subjected to a great amount of variability if sensor equipment, placement, and location procedures are not standardized. Variability was reduced by using the recommended procedures of the SENIAM (Surface Electromyography for Non-Invasive Assessment of Muscles) guidelines^27^. The SENIAM project formulated European recommendations for sensor placement procedures and signal processing. Sensor equipment consisted of polymer Ag/AgCl coated circular gel electrodes (Covidien Kendall^TM^, 30 mm × 24 mm) with a bipolar sensor (Zerowire, ©Cometa, Barregio, Italy). Electrodes were placed on dry, degreased (with diethylether), and shaved skin, with a 20 mm inter-electrode distance and in the direction of the muscle fibers. The placement was performed by the same experienced researcher (TVC) in all participants and performed in a lying position for all muscles except for the erector spinae and hamstrings which were placed in a sitting and prone position, respectively. The electrodes were located as described by SENIAM, in the longitudinal direction halfway of the motor endplate and tendon. The participants were asked to contract the muscle to verify the correctness of the location. Wireless sensors were placed so that no stretch was applied on cables and were firmly attached with double-sided tape and tape over the sensor. The signal-to-noise ratio was checked in the Vicon software by asking participants to maximally contract each muscle (if possible) to ensure clear EMG-recordings. All procedures are in agreement with the SENIAM guidelines ensuring reliable and accurate EMG data collection.

## Usage Notes

All anonymized data records are available at Figshare^32^, including both the source *C3D* files (https://www.c3d.org) and can be easily read by different motion capture programs (eg. Vicon Nexus, Qualisys), biomechanical analysis software (eg. Visual3D), and programming software (eg. Matlab, Python, C++ using “read C3D toolbox” for Matlab or other available open source toolboxes^42^). This format supports the combination of different data types and length.

The post-processed dataset including all able-bodied or post-stroke participants in *MAT-*file format. For more details on folder- and filenames, see data records section. For an explanation of the *MAT-*file structure see file “*MATdatafiles_description_v1.3_LST*” and the number of strides with good kinematic and kinetic data (“*NrStrides*”), and a visual presentation (*MAT-figure* and*.png*) of the selected variables.

The *MAT-*files can be easily opened and used with MATLAB, but can also be opened in other coding software with some additional steps and toolboxes (e.g., Python). The authors have created a GitHub page where a MatToPy tool is available, this tool imports of Matlab data into Python to support further analysis in Python. The script can be accessed through this link: https://github.com/LizSloot/Readin_MatlabStruct_toPython.git. Authors made this script available through GitHub such that further optimization of the script is possible. Furthermore, computation of the 3D kinematics, kinetics and muscular activity from the provided data can be performed by the freely available code toolboxes proposed by Dumas, Too and the Advance Gait Workflow from the Vicon website (https://nl.mathworks.com/matlabcentral/fileexchange/58021-3d-kinematics-and-inverse-dynamics; https://www.mathworks.com/matlabcentral/fileexchange/71514-emg-feature-extraction-toolbox?s_tid=srchtitle; https://www.vicon.com/software/models-and-scripts/nexus-advanced-gait-workflow/?section=downloads). Bespoke Matlab code to create plots as those shown in the Supplementary data is provided with the Supplementary files.

### Supplementary information

Supplementary tables

## Data Availability

See usage notes for information on code availability to compute or process the available data.

## References

[1] Baker R (2006). Gait analysis methods in rehabilitation. J Neuroeng Rehabil.

[2] Bruening DA, Baird AR, Weaver KJ, Rasmussen AT (2020). Whole body kinematic sex differences persist across non-dimensional gait speeds. PLoS One.

[3] Moore JK, Hnat SK, van den Bogert AJ (2015). An elaborate data set on human gait and the effect of mechanical perturbations. PeerJ.

[4] Winter, D. A. *Biomechanics and motor control of human movement*. 4th edn, (Wiley, 2009).

[5] Wang, Y. & Srinivasan, M. Stepping in the direction of the fall: the next foot placement can be predicted from current upper body state in steady-state walking. *Biol Lett* **10**, 10.1098/rsbl.2014.0405 (2014). 10.1098/rsbl.2014.0405 PMC4190959 25252834

[6] Fukuchi CA, Fukuchi RK, Duarte M (2018). A public dataset of overground and treadmill walking kinematics and kinetics in healthy individuals. PeerJ.

[7] van den Bogert AJ, Geijtenbeek T, Even-Zohar O, Steenbrink F, Hardin EC (2013). A real-time system for biomechanical analysis of human movement and muscle function. Med Biol Eng Comput.

[8] Bertaux A (2022). Gait analysis dataset of healthy volunteers and patients before and 6 months after total hip arthroplasty. Sci Data.

[9] David, P. F., David, R. C., Juan, M. C. & Diego, T. Human Locomotion Databases. A Systematic Review. *IEEE J Biomed Health Inform* PP, 10.1109/JBHI.2023.3311677 (2023). 10.1109/JBHI.2023.3311677 37665701

[10] Lencioni T, Carpinella I, Rabuffetti M, Marzegan A, Ferrarin M (2019). Human kinematic, kinetic and EMG data during different walking and stair ascending and descending tasks. Sci Data.

[11] Schreiber C, Moissenet F (2019). A multimodal dataset of human gait at different walking speeds established on injury-free adult participants. Sci Data.

[12] http://mocap.cs.cmu.edu, H. H. J. C. g. l. m. c. d. h. m. c. c. e.

[13] Murray MP, Kory RC, Clarkson BH (1969). Walking patterns in healthy old men. J Gerontol.

[14] Ko SU, Hausdorff JM, Ferrucci L (2010). Age-associated differences in the gait pattern changes of older adults during fast-speed and fatigue conditions: results from the Baltimore longitudinal study of ageing. Age Ageing.

[15] Jerome GJ (2015). Gait characteristics associated with walking speed decline in older adults: results from the Baltimore Longitudinal Study of Aging. Arch Gerontol Geriatr.

[16] Sloot LH (2021). Decline in gait propulsion in older adults over age decades. Gait Posture.

[17] Van Criekinge T (2018). Age-related differences in muscle activity patterns during walking in healthy individuals. J Electromyogr Kinesiol.

[18] Serrao M (2018). Dataset on gait patterns in degenerative neurological diseases. Data Brief.

[19] Van Criekinge T, Hallemans A, Van de Walle P, Sloot LH (2021). Age-related changes in trunk kinematics and mechanical work during gait. Gait & Posture.

[20] Herssens N (2020). An investigation of the spatio-temporal parameters of gait and margins of stability throughout adulthood. J R Soc Interface.

[21] Sloot L, Millard M, Mombaur K, Hallemans A, van Criekinge T (2021). Ageing does not affect the dynamic balance margins of gait. Gait & Posture.

[22] Van Criekinge T (2020). Trunk biomechanics during walking after sub-acute stroke and its relation to lower limb impairments. Clin Biomech (Bristol, Avon).

[23] Verheyden G (2005). Discriminant ability of the Trunk Impairment Scale: A comparison between stroke patients and healthy individuals. Disabil Rehabil.

[24] Mehrholz J, Wagner K, Rutte K, Meissner D, Pohl M (2007). Predictive validity and responsiveness of the functional ambulation category in hemiparetic patients after stroke. Arch Phys Med Rehabil.

[25] Verheyden G (2004). The Trunk Impairment Scale: a new tool to measure motor impairment of the trunk after stroke. Clin Rehabil.

[26] Tinetti ME (1986). Performance-oriented assessment of mobility problems in elderly patients. J Am Geriatr Soc.

[27] Stegeman, D. H., Hermie Standards for surface electromyography: The European project Surface EMG for non-invasive assessment of muscles (SENIAM) (2007).

[28] Davis RB, Õunpuu S, Tyburski D, Gage JR (1991). A gait analysis data collection and reduction technique. Human Movement Science.

[29] Grood ES, Suntay WJ (1983). A joint coordinate system for the clinical description of three-dimensional motions: application to the knee. J Biomech Eng.

[30] Schwartz MH, Trost JP, Wervey RA (2004). Measurement and management of errors in quantitative gait data. Gait & Posture.

[31] Sousa, A. & Tavares, J. Surface electromyographic amplitude normalization methods: A review 85–102 (2012).

[32] Van Criekinge (2023). Figshare..

[33] Kadaba MP (1989). Repeatability of kinematic, kinetic, and electromyographic data in normal adult gait. J Orthop Res.

[34] Wilken JM, Rodriguez KM, Brawner M, Darter BJ (2012). Reliability and Minimal Detectible Change values for gait kinematics and kinetics in healthy adults. Gait Posture.

[35] Bates AV, McGregor AH, Alexander CM (2016). Reliability and minimal detectable change of gait kinematics in people who are hypermobile. Gait Posture.

[36] Fernandes R, Armada-da-Silva P, Pool-Goudzwaard AL, Moniz-Pereira V, Veloso AP (2016). Three dimensional multi-segmental trunk kinematics and kinetics during gait: Test-retest reliability and minimal detectable change. Gait Posture.

[37] Merriaux, P., Dupuis, Y., Boutteau, R., Vasseur, P. & Savatier, X. A Study of Vicon System Positioning Performance. *Sensors (Basel)* **17**, 10.3390/s17071591 (2017). 10.3390/s17071591 PMC5551098 28686213

[38] Gorton GE, Hebert DA, Gannotti ME (2009). Assessment of the kinematic variability among 12 motion analysis laboratories. Gait Posture.

[39] Ferrari A (2008). Quantitative comparison of five current protocols in gait analysis. Gait Posture.

[40] Kainz H (2017). Reliability of four models for clinical gait analysis. Gait Posture.

[41] Molina-Rueda, F. *et al*. Test-Retest Reliability of a Conventional Gait Model for Registering Joint Angles during Initial Contact and Toe-Off in Healthy Subjects. *Int J Environ Res Public Health* **18**, 10.3390/ijerph18031343 (2021). 10.3390/ijerph18031343 PMC7908319 33540795

[42] Pariterre. ezc3d (https://github.com/pyomeca/ezc3d), *GitHub*. Retrieved February 8, 2023 (2023).

