# Supplementary material for: A full-body motion capture gait dataset of 138 able-bodied adults across the life span and 50 stroke survivors
Source: Sci Data. 2023 Dec 1;10:852. doi: 10.1038/s41597-023-02767-y (PMC10692332; doi:10.1038/s41597-023-02767-y)
Supplement: Supplementary file 1 — Supplementary tables [file 41597_2023_2767_MOESM1_ESM.pdf]

**Supplementary Table 1. Subject characteristics of able-bodied adults**

| ID | Age (y) | Sex (M/F) | Body mass (kg) | Height (mm) | Leg Length (mm) | ID | Age (y) | Sex (M/F) | Body mass (kg) | Height (mm) | Leg Length (mm) |
|---|---|---|---|---|---|---|---|---|---|---|---|
| **SUBJ1** | 86 | M | 64 | 1580 | 850 | **SUBJ52** | 58 | M | 79 | 1715 | 920 |
| **SUBJ2** | 85 | F | 78 | 1500 | 840 | **SUBJ53** | 58 | M | 97 | 1835 | 990 |
| **SUBJ3** | 85 | F | 69 | 1510 | 880 | **SUBJ54** | 57 | F | 58 | 1640 | 870 |
| **SUBJ4** | 84 | M | 70 | 1625 | 950 | **SUBJ55** | 57 | F | 62 | 1580 | 870 |
| **SUBJ5** | 84 | F | 50 | 1450 | 810 | **SUBJ56** | 55 | F | 58 | 1635 | 820 |
| **SUBJ6** | 83 | M | 80 | 1695 | 970 | **SUBJ57** | 55 | M | 67 | 1685 | 880 |
| **SUBJ7** | 82 | F | 62 | 1555 | 840 | **SUBJ58** | 55 | M | 66 | 1795 | 930 |
| **SUBJ8** | 82 | F | 72 | 1600 | 820 | **SUBJ59** | 54 | F | 57 | 1675 | 860 |
| **SUBJ9** | 82 | M | 87 | 1710 | 920 | **SUBJ60** | 53 | M | 90 | 1780 | 940 |
| **SUBJ10** | 81 | M | 73 | 1620 | 870 | **SUBJ61** | 53 | M | 89 | 1770 | 960 |
| **SUBJ11** | 81 | M | 76 | 1700 | 910 | **SUBJ62** | 53 | M | 69 | 1765 | 900 |
| **SUBJ12** | 80 | M | 101 | 1890 | 1070 | **SUBJ63** | 53 | F | 72 | 1655 | 880 |
| **SUBJ13** | 80 | F | 70 | 1540 | 870 | **SUBJ64** | 52 | F | 53 | 1580 | 830 |
| **SUBJ14** | 80 | M | 87 | 1755 | 920 | **SUBJ65** | 52 | F | 62 | 1560 | 840 |
| **SUBJ15** | 83 | F | 74 | 1530 | 820 | **SUBJ66** | 55 | M | 86 | 1755 | 910 |
| **SUBJ16** | 79 | F | 74 | 1610 | 860 | **SUBJ67** | 55 | F | 48 | 1565 | 831 |
| **SUBJ17** | 77 | M | 86 | 1750 | 950 | **SUBJ68** | 51 | F | 57 | 1630 | 840 |
| **SUBJ18** | 77 | M | 90 | 1770 | 940 | **SUBJ69** | 50 | F | 51 | 1685 | 890 |
| **SUBJ19** | 77 | M | 88 | 1830 | 1030 | **SUBJ70** | 54 | F | 67 | 1660 | 890 |
| **SUBJ20** | 76 | M | 79 | 1750 | 970 | **SUBJ71** | 50 | M | 80 | 1885 | 1050 |
| **SUBJ21** | 75 | F | 78 | 1420 | 660 | **SUBJ72** | 49 | M | 81 | 1670 | 860 |
| **SUBJ22** | 79 | M | 83 | 1645 | 898 | **SUBJ73** | 49 | F | 69 | 1680 | 900 |
| **SUBJ23** | 75 | M | 89 | 1665 | 880 | **SUBJ74** | 49 | M | 80 | 1815 | 950 |
| **SUBJ24** | 74 | F | 72 | 1585 | 850 | **SUBJ75** | 48 | F | 67 | 1600 | 860 |
| **SUBJ25** | 74 | F | 83 | 1545 | 810 | **SUBJ76** | 48 | M | 97 | 1780 | 960 |
| **SUBJ26** | 73 | F | 64 | 1605 | 860 | **SUBJ77** | 48 | M | 80 | 1880 | 990 |
| **SUBJ27** | 73 | F | 61 | 1595 | 870 | **SUBJ78** | 51 | F | 64 | 1605 | 870 |
| **SUBJ28** | 72 | F | 69 | 1605 | 860 | **SUBJ79** | 46 | F | 48 | 1525 | 800 |
| **SUBJ29** | 72 | F | 75 | 1560 | 880 | **SUBJ80** | 50 | M | 72 | 1730 | 875 |
| **SUBJ30** | 72 | F | 61 | 1585 | 870 | **SUBJ81** | 45 | M | 77 | 1750 | 930 |
| **SUBJ31** | 71 | M | 65 | 1610 | 830 | **SUBJ82** | 45 | F | 70 | 1915 | 830 |
| **SUBJ32** | 71 | M | 83 | 1725 | 950 | **SUBJ83** | 44 | F | 55 | 1475 | 760 |
| **SUBJ33** | 70 | M | 82 | 1795 | 995 | **SUBJ84** | 44 | F | 70 | 1585 | 850 |
| **SUBJ34** | 69 | M | 76 | 1655 | 890 | **SUBJ85** | 44 | M | 79 | 1675 | 910 |
| **SUBJ35** | 67 | M | 92 | 1830 | 970 | **SUBJ86** | 44 | F | 81 | 1655 | 880 |
| **SUBJ36** | 67 | M | 74 | 1690 | 930 | **SUBJ87** | 44 | M | 100 | 1775 | 950 |
| **SUBJ37** | 66 | F | 55 | 1615 | 860 | **SUBJ88** | 44 | F | 94 | 1750 | 930 |
| **SUBJ38** | 66 | F | 57 | 1635 | 870 | **SUBJ89** | 43 | M | 74 | 1755 | 950 |
| **SUBJ39** | 65 | F | 69 | 1645 | 890 | **SUBJ90** | 42 | F | 50 | 1535 | 780 |
| **SUBJ40** | 65 | M | 95 | 1750 | 930 | **SUBJ91** | 41 | F | 58 | 1535 | 800 |
| **SUBJ41** | 64 | F | 75 | 1580 | 900 | **SUBJ92** | 40 | F | 109 | 1720 | 880 |
| **SUBJ42** | 64 | F | 82 | 1550 | 840 | **SUBJ93** | 39 | F | 73 | 1665 | 900 |
| **SUBJ43** | 62 | M | 95 | 1770 | 960 | **SUBJ94** | 42 | M | 79 | 1725 | 935 |
| **SUBJ44** | 62 | F | 80 | 1565 | 860 | **SUBJ95** | 37 | M | 103 | 1920 | 1020 |
| **SUBJ45** | 61 | F | 79 | 1615 | 880 | **SUBJ96** | 36 | M | 157 | 1830 | 950 |
| **SUBJ46** | 60 | F | 66 | 1580 | 860 | **SUBJ97** | 36 | M | 65 | 1735 | 950 |
| **SUBJ47** | 60 | M | 69 | 1710 | 960 | **SUBJ98** | 36 | M | 76 | 1765 | 970 |
| **SUBJ48** | 60 | M | 59 | 1740 | 880 | **SUBJ99** | 34 | F | 62 | 1700 | 940 |
| **SUBJ49** | 60 | F | 85 | 1645 | 910 | **SUBJ100** | 34 | M | 78 | 1820 | 980 |
| **SUBJ50** | 59 | F | 62 | 1515 | 800 | **SUBJ101** | 34 | F | 60 | 1715 | 900 |
| **SUBJ51** | 59 | F | 64 | 1660 | 870 | **SUBJ102** | 33 | F | 59 | 1575 | 830 |

| | | | | | | | | | | | |
|---|---|---|---|---|---|---|---|---|---|---|---|
| **SUBJ103** | 33 | F | 70 | 1605 | 890 | **SUBJ121** | 25 | F | 65 | 1705 | 900 |
| **SUBJ104** | 33 | M | 84 | 1795 | 920 | **SUBJ122** | 25 | F | 67 | 1645 | 880 |
| **SUBJ105** | 33 | M | 96 | 1910 | 998 | **SUBJ123** | 24 | F | 63 | 1735 | 930 |
| **SUBJ106** | 32 | F | 68 | 1775 | 950 | **SUBJ124** | 24 | M | 76 | 1765 | 910 |
| **SUBJ107** | 32 | M | 77 | 1780 | 900 | **SUBJ125** | 24 | M | 82 | 1850 | 1030 |
| **SUBJ108** | 36 | F | 71 | 1645 | 890 | **SUBJ126** | 24 | M | 84 | 1840 | 1020 |
| **SUBJ109** | 31 | F | 58 | 1675 | 880 | **SUBJ127** | 28 | M | 102 | 1710 | 900 |
| **SUBJ110** | 30 | F | 65 | 1670 | 870 | **SUBJ128** | 24 | F | 56 | 1580 | 850 |
| **SUBJ111** | 29 | M | 115 | 1730 | 940 | **SUBJ129** | 25 | F | 67 | 1640 | 885 |
| **SUBJ112** | 28 | F | 71 | 1690 | 940 | **SUBJ130** | 21 | F | 65 | 1590 | 820 |
| **SUBJ113** | 27 | M | 85 | 1770 | 930 | **SUBJ131** | 21 | F | 66 | 1670 | 880 |
| **SUBJ114** | 27 | M | 76 | 1705 | 900 | **SUBJ132** | 24 | F | 65 | 1630 | 850 |
| **SUBJ115** | 27 | M | 87 | 1805 | 960 | **SUBJ133** | 23 | M | 64 | 1730 | 910 |
| **SUBJ116** | 30 | M | 89 | 1770 | 955 | **SUBJ134** | 23 | F | 65 | 1625 | 915 |
| **SUBJ117** | 26 | F | 68 | 1780 | 920 | **SUBJ135** | 22 | M | 104 | 1810 | 970 |
| **SUBJ118** | 30 | M | 65 | 1830 | 975 | **SUBJ136** | 22 | F | 61 | 1625 | 830 |
| **SUBJ119** | 30 | M | 76 | 1850 | 1005 | **SUBJ137** | 22 | F | 78 | 1700 | 880 |
| **SUBJ120** | 25 | F | 58 | 1710 | 890 | **SUBJ138** | 21 | M | 68 | 1675 | 880 |
| **ID: identification number, y: years, M: male, F: female, kg: kilograms, mm: millimetre** | | | | | | | | | | | |

## Supplementary Table 2. Subject characteristics of stroke survivors

| ID | Age (y) | Gender (M/F) | Weight (kg) | Height (mm) | Leg Length (mm) | Time post stroke (days) | Lesion location (L/R) | Type of stroke (I/H) | FAC | Tinetti POMA | TIS |
|---|---|---|---|---|---|---|---|---|---|---|---|
| **TVC03** | 58 | M | 67 | 1710 | 870 | 114 | L | I | 3 | 23 | 16 |
| **TVC04** | 77 | F | 68 | 1585 | 880 | 37 | R | I | 4 | 27 | 19 |
| **TVC05** | 47 | F | 65 | 1600 | 840 | 104 | R | I | 2 | 14 | 13 |
| **TVC06** | 49 | M | 106 | 1755 | 950 | 57 | R | H | 3 | 21 | 18 |
| **TVC08** | 71 | M | 88 | 1740 | 880 | 64 | R | I | 3 | 15 | 17 |
| **TVC09** | 81 | M | 78 | 1665 | 940 | 139 | R | H | 4 | 26 | 14 |
| **TVC10** | 67 | F | 52 | 1500 | 810 | 64 | R | I | 2 | 21 | 12 |
| **TVC11** | 55 | F | 60 | 1800 | 960 | 14 | R | I | 3 | 21 | 18 |
| **TVC12** | 71 | M | 77 | 1650 | 880 | 62 | L | H | 3 | 19 | 13 |
| **TVC13** | 77 | M | 83 | 1800 | 910 | 32 | R | I | 2 | 18 | 14 |
| **TVC14** | 73 | M | 82 | 1785 | 970 | 59 | R | I | 2 | 12 | 13 |
| **TVC15** | 72 | M | 80 | 1700 | 900 | 18 | L | I | 5 | 26 | 16 |
| **TVC16** | 54 | F | 58 | 1610 | 860 | 21 | R | I | 2 | 11 | 10 |
| **TVC17** | 70 | M | 80 | 1690 | 900 | 45 | L | I | 3 | 20 | 17 |
| **TVC19** | 60 | M | 71 | 1790 | 930 | 60 | R | I | 3 | 17 | 15 |
| **TVC20** | 44 | M | 59 | 1720 | 940 | 91 | L | I | 5 | 28 | 15 |
| **TVC21** | 50 | F | 72 | 1730 | 930 | 106 | R | I | 2 | 13 | 15 |
| **TVC22** | 58 | F | 83 | 1700 | 910 | 46 | L | I | 3 | 19 | 16 |
| **TVC23** | 39 | F | 70 | 1670 | 840 | 60 | R | H | 3 | 16 | 17 |
| **TVC24** | 77 | M | 83 | 1690 | 850 | 63 | R | I | 3 | 21 | 15 |
| **TVC25** | 84 | F | 84 | 1580 | 820 | 31 | L | I | 4 | 24 | 9 |
| **TVC26** | 68 | F | 64 | 1650 | 880 | 61 | L | H | 3 | 15 | 12 |
| **TVC27** | 69 | M | 69 | 1700 | 890 | 39 | L | I | 3 | 21 | 14 |
| **TVC28** | 62 | M | 95 | 1800 | 920 | 26 | R | I | 2 | 17 | 8 |
| **TVC29** | 72 | F | 58 | 1700 | 820 | 31 | L | I | 2 | 7 | 12 |
| **TVC30** | 72 | M | 99 | 1680 | 810 | 20 | L | I | 3 | 18 | 8 |
| **TVC32** | 52 | M | 80 | 1790 | 940 | 25 | R | I | 3 | 20 | 14 |
| **TVC33** | 63 | M | 85 | 1890 | 960 | 21 | L | I | 2 | 14 | 15 |
| **TVC34** | 80 | M | 76 | 1670 | 880 | 41 | R | I | 3 | 23 | 15 |
| **TVC35** | 72 | M | 54 | 1670 | 810 | 24 | R | I | 2 | 12 | 13 |
| **TVC36** | 41 | M | 65 | 1660 | 840 | 50 | R | H | 2 | 10 | 9 |
| **TVC37** | 72 | M | 53 | 1720 | 880 | 45 | R | I | 3 | 21 | 12 |
| **TVC38** | 72 | M | 61 | 1730 | 890 | 65 | R | I | 2 | 8 | 11 |
| **TVC39** | 65 | M | 86 | 1790 | 900 | 42 | L | I | 2 | 13 | 7 |
| **TVC40** | 19 | M | 67 | 1810 | 940 | 106 | L | H | 4 | 23 | 17 |
| **TVC41** | 53 | F | 50 | 1530 | 790 | 90 | L | I | 3 | 8 | 10 |
| **TVC42** | 75 | M | 54 | 1720 | 870 | 48 | R | I | 2 | 13 | 9 |
| **TVC43** | 84 | M | 80 | 1710 | 900 | 38 | R | I | 4 | 27 | 13 |
| **TVC46** | 52 | M | 68 | 1830 | 930 | 31 | R | I | 5 | 28 | 14 |
| **TVC47** | 81 | F | 62 | 1630 | 810 | 36 | L | I | 4 | 28 | 19 |
| **TVC48** | 75 | M | 51 | 1690 | 824 | 50 | R | I | 2 | 6 | 13 |
| **TVC49** | 73 | M | 70 | 1700 | 870 | 36 | R | I | 3 | 15 | 9 |
| **TVC51** | 46 | M | 81 | 1640 | 840 | 104 | R | I | 3 | 22 | 14 |
| **TVC52** | 62 | M | 73 | 1650 | 870 | 42 | R | I | 5 | 28 | 18 |
| **TVC53** | 76 | M | 63 | 1800 | 945 | 53 | R | H | 3 | 21 | 12 |
| **TVC54** | 85 | M | 65 | 1800 | 860 | 32 | R | I | 2 | 12 | 10 |
| **TVC55** | 75 | F | 70 | 1730 | 850 | 67 | R | I | 2 | 20 | 14 |
| **TVC57** | 55 | M | 104 | 1800 | 900 | 26 | L | H | 4 | 24 | 20 |
| **TVC58** | 59 | F | 76 | 1640 | 840 | 41 | R | H | 2 | 25 | 14 |
| **TVC60** | 46 | F | 55 | 1630 | 850 | 86 | L | H | 4 | 26 | 18 |

**ID: identification number, y: years, M: male, F: female, kg: kilograms mm: millimetre, L: left, R: right, I: ischeamic, H: hemorrhagic, FAC: Functional Amublation Category, Tinetti POMA: Tinetti Performance Oriented Mobility Test, TIS: Trunk Impairment Scale**

**Supplementary Table 3. EMG and force plate recordings for able-bodied adults**

*Legenda* – L: left, R; right, FP: force plate, EMG: electromyography, CP: cross plate, (1): trial 1, (2): trial 2, (3): trial 3

| ID | EMG | FP 1 | FP 2 | FP 3 | FP 4 | ID | EMG | FP 1 | FP 2 | FP 3 | FP 4 |
|---|---|---|---|---|---|---|---|---|---|---|---|
| **SUBJ 1** | Yes | R (1)<br>R (2) | L (1)<br>L (2) | X | R (1)<br>R (2) | **SUBJ 18** | Yes | R (2) | L CP (2)<br>L (3) | X | L CP (2) |
| **SUBJ 2** | Yes | R (1)<br>L (2) | L (1)<br>R (2) | X | R (1) | **SUBJ 19** | Yes | R (1)<br>R (3) | L (1)<br>L (2) | X | R (2) |
| **SUBJ 3** | Yes | L (2)<br>L (3) | R (3) | X | L (3) | **SUBJ 20** | Yes | R (1)<br>R (2)<br>R (3) | L (1)<br>L (2)<br>L (3) | X | R (1)<br>R (2)<br>R (3) |
| **SUBJ 4** | Yes | R (1)<br>R (2) | L (2) | X | R (2) | **SUBJ 21** | No | R (1)<br>L (2) | R (2) | R (4) | L (2)<br>L (4) |
| **SUBJ 5** | Yes | R (1)<br>R (2) | L (1)<br>L (2)<br>R (3) | X | R (2)<br>L (3) | **SUBJ 22** | Yes | R (1)<br>R (2)<br>R (3) | L (1)<br>L (2) | X | R (1)<br>R (2) |
| **SUBJ 6** | Yes | L (3) | R (1)<br>R (3) | X | L (1)<br>L (2) | **SUBJ 23** | No | L (2)<br>R (3) | R (2)<br>L (3) | X | L (2)<br>R (3) |
| **SUBJ 7** | Yes | R (3) | L (2)<br>L (3) | X | R (2)<br>R (3) | **SUBJ 24** | Yes | R (1)<br>R (2)<br>R (3) | L (1)<br>L (2)<br>L (3) | X | R (1)<br>R (2)<br>R (3) |
| **SUBJ 8** | Yes | R (1)<br>R (2)<br>R (3) | L (1)<br>L (2)<br>L (3) | X | R (1)<br>R (2)<br>R (3) | **SUBJ 25** | Yes | R (1)<br>R (2)<br>R (3) | L (1)<br>L (2)<br>L (3) | X | R (1)<br>R (2)<br>R (3) |
| **SUBJ 9** | Yes | L CP (2)<br>R (3) | R (1)<br>R CP (2)<br>R CP(3) | L CP (2) | L (1)<br>R CP (2)<br>R CP (3) | **SUBJ 26** | Yes | R (1)<br>L (2) | L (1)<br>R (2)<br>R (3) | X | L (3) |
| **SUBJ 10** | Yes | R (1) | L (2)<br>R (3) | X | X | **SUBJ 27** | Yes | R (3) | L (3) | X | R (3) |
| **SUBJ 11** | Yes | R (1) | R (3) | L (1) | R (1)<br>L (2)<br>L (3) | **SUBJ 28** | Yes | R (1)<br>R (2)<br>L (3) | L (1)<br>L (2)<br>R (3) | X | R (2)<br>L (3) |
| **SUBJ 12** | Yes | L (2)<br>R (3) | R CP (1)<br>R (2) | R CP (1) | L (1) | **SUBJ 29** | Yes | L (1)<br>L (2)<br>R (3) | R (1) | X | X |
| **SUBJ 13** | Yes | L (2)<br>L (3) | R (2)<br>R (3) | X | R (1)<br>L (2)<br>L (3) | **SUBJ 30** | Yes | L (3) | X | X | R (1)<br>R (2) |
| **SUBJ 14** | Yes | R (1) | L (1)<br>L (3) | X | R (1)<br>R (2)<br>R (3) | **SUBJ 31** | Yes | L (3) | L (1)<br>R (3) | X | R (2) |
| **SUBJ 15** | Yes | L (1)<br>R (2)<br>R (3) | R (1)<br>L (2)<br>L (3) | X | L (1)<br>R (2)<br>R (3) | **SUBJ 32** | Yes | R (2)<br>R (4) | L (2) | X | L (1) |
| **SUBJ 16** | Yes | L (2)<br>R (4) | R CP (2)<br>L (4) | X | R CP (2)<br>L (3) | **SUBJ 33** | Yes | R (2)<br>L (3) | L (2) | X | L (1)<br>R (2) |
| **SUBJ 17** | Yes | R (2)<br>R (3) | L (1)<br>L CP (2)<br>L (3) | X | R (1)<br>L CP (2) | **SUBJ 34** | Yes | R CP (1)<br>R (2)<br>L (3) | L CP (1)<br>L CP (2)<br>R CP (3) | R CP (1) | L CP (1)<br>L CP (2)<br>R CP (3) |
| **SUBJ 35** | Yes | R CP (3)<br>L (4) | L (3)<br>R (4) | R CP(3) | R (1) | **SUBJ 53** | Yes | L CP (1)<br>L CP (2)<br>L (3) | L CP (1)<br>L CP (2)<br>R CP (3) | X | R (1)<br>R (2)<br>R CP (3) |
| **SUBJ 36** | Yes | L (2)<br>R (3) | L (1)<br>L CP (3) | X | R (1)<br>L CP (3) | **SUBJ 54** | Yes | R (1) | L CP (1)<br>L (2) | X | L CP (1) |

| | | | | | | | | | | | |
|---|---|---|---|---|---|---|---|---|---|---|---|
| | | | | | | | | R CP (3) | R CP (3) | | L (3) |
| **SUBJ 37** | Yes | X | L (1) | X | R (1)<br>R (2)<br>R (3) | **SUBJ 55** | Yes | L (1)<br>L (2)<br>R (3) | R (1)<br>L (3) | X | L (1) |
| **SUBJ 38** | Yes | R (1)<br>L (2)<br>R (3) | L (1)<br>L (3) | X | X | **SUBJ 56** | Yes | R (1)<br>R CP (2)<br>L (3)<br>R CP (4) | L CP (1)<br>R CP (2)<br>R CP (3)<br>R CP (4) | X | L CP (1)<br>L (4)<br>R CP (3)<br>L (4) |
| **SUBJ 39** | Yes | L (1)<br>R CP (2) | R CP (2) | X | L (2) | **SUBJ 57** | Yes | L (1) | R (1) | X | L (1)<br>R (2)<br>L (3) |
| **SUBJ 40** | Yes | R (3) | L (3) | X | R (2)<br>R (3) | **SUBJ 58** | Yes | L CP (2)<br>L CP (3) | L (1)<br>L CP (2)<br>L CP (3) | X | R (3) |
| **SUBJ 41** | Yes | L (2)<br>L CP (3) | R (1)<br>R (2)<br>L CP (3) | L CP (3) | L (1)<br>L (2) | **SUBJ 59** | Yes | L (1)<br>L (2) | L (3) | X | R (3) |
| **SUBJ 42** | Yes | R (1)<br>L (2) | L (1)<br>R (3) | X | R (1)<br>L (3) | **SUBJ 60** | Yes | R CP (2)<br>R CP (4) | R (1)<br>R CP (2)<br>R (3)<br>R CP (4) | X | L (2)<br>L (4) |
| **SUBJ 43** | Yes | R (3) | X | X | L (1)<br>L (2) | **SUBJ 61** | Yes | L (1)<br>L (2) | R (1) | X | L (1) |
| **SUBJ 44** | Yes | R CP(4) | R CP (4) | X | R (1)<br>L (4)<br>L (5) | **SUBJ 62** | Yes | L (1)<br>L (2)<br>L (3) | R (3) | X | L (3) |
| **SUBJ 45** | Yes | R (1)<br>R (2)<br>R CP (3) | L CP (1)<br>L (2)<br>R CP (3) | X | L CP (1)<br>L (3) | **SUBJ 63** | Yes | | L (1)<br>L (2)<br>L (3) | X | R (1)<br>R (3) |
| **SUBJ 46** | Yes | R CP (1)<br>R (2) | R CP (1)<br>L CP (2) | X | L (1)<br>L CP (2) | **SUBJ 64** | Yes | L (1)<br>R CP (2)<br>L CP (3) | R CP (1)<br>R CP (2)<br>L CP (3) | X | R CP (1)<br>L (2)<br>R (3) |
| **SUBJ 47** | Yes | L (1)<br>R (2)<br>L (3) | R (1)<br>L (2) | X | X | **SUBJ 65** | Yes | L (1) | L (3) | X | R (2) |
| **SUBJ 48** | No | R (1)<br>R (3) | L (1)<br>L (3) | X | R (1)<br>R (3) | **SUBJ 66** | Yes | R (1)<br>R (3)<br>L CP (4) | R (1)<br>L CP (3)<br>L CP (4) | X | L (1)<br>L CP (3)<br>R (4) |
| **SUBJ 49** | Yes | R (2)<br>L (3) | R (1)<br>L (2)<br>R (3) | X | L (1)<br>R (2)<br>L (3) | **SUBJ 67** | Yes | R (1)<br>R (2)<br>R (3) | L (1)<br>L (2) | X | R (1) |
| **SUBJ 50** | Yes | L (1)<br>L (2)<br>L (3) | R (2)<br>R (3) | X | L (2)<br>L (3) | **SUBJ 68** | Yes | R CP (1)<br>R CP (2)<br>R (3) | R CP (1)<br>R CP (2)<br>L CP (3) | X | L (1)<br>L (2)<br>L CP (3) |
| **SUBJ 51** | Yes | R CP (1)<br>L CP (3) | R CP (1)<br>R (2)<br>L CP (3) | X | L (1)<br>R (3) | **SUBJ 69** | Yes | L (2) | L (1)<br>R CP (2)<br>R (3) | X | R (1)<br>R CP (2)<br>L (3) |
| **SUBJ 52** | Yes | L (1)<br>R (2)<br>R (3) | L (2)<br>L (3) | X | R (2)<br>R (3) | **SUBJ 70** | Yes | R (1)<br>L (2)<br>L (2) | R (2) | X | X |
| **SUBJ 71** | Yes | L CP (1)<br>L (3) | L CP (1)<br>L CP (2)<br>R CP(3) | X | L CP (2)<br>R CP (3) | **SUBJ 89** | Yes | R (1) | L (1) | X | L (2) |
| **SUBJ 72** | No | R (1)<br>R (2) | L CP (1)<br>L (2) | | L CP (1) | **SUBJ 90** | Yes | R (1)<br>L CP (2) | L CP (1)<br>L CP (2) | X | L CP (1)<br>R (2) |

| | | | | | | | | | | | |
|---|---|---|---|---|---|---|---|---|---|---|---|
| | | R CP (3) | L CP (3) | R CP (3) | L CP (3) | | | L (3)<br>R CP (4) | R CP (4) | | L (4) |
| **SUBJ 73** | Yes | L (2)<br>R (3) | L (1)<br>R (2)<br>L (3) | X | L (2)<br>R (3) | **SUBJ 91** | Yes | R (1)<br>R (2) | L (1)<br>L (2) | X | R (1) |
| **SUBJ 74** | No | R (2)<br>R (3) | L CP (2)<br>L CP (3)<br>R (4) | X | R (1)<br>L CP (2)<br>L CP (3)<br>L (4) | **SUBJ 92** | Yes | R (2) | L (3) | X | R (3) |
| **SUBJ 75** | Yes | L (1)<br>L (2)<br>R (3) | R (1)<br>R (2)<br>L (3) | X | L (1)<br>L (2)<br>R (3) | **SUBJ 93** | Yes | R (3) | L (1) | X | R (1) |
| **SUBJ 76** | Yes | R (1)<br>R (2)<br>L (3) | L (1)<br>R (3) | X | X | **SUBJ 94** | Yes | R (4) | L (4) | X | L (3)<br>R (4) |
| **SUBJ 77** | Yes | L (1)<br>L (2) | R (2) | X | X | **SUBJ 95** | Yes | L (2)<br>R (3) | R (1)<br>L (3) | X | L (1)<br>L (3) |
| **SUBJ 78** | Yes | L (2) | L (1)<br>R (2)<br>L (3) | X | R (1)<br>L (2)<br>R (3) | **SUBJ 96** | Yes | L (1)<br>L CP (2)<br>L (3) | R (1)<br>L CP (2)<br>R CP (3) | X | R (2)<br>R CP (3) |
| **SUBJ 79** | Yes | L (1) | R (1)<br>L (2)<br>L (3) | X | R (2)<br>R (3) | **SUBJ 97** | Yes | L CP (1)<br>L CP (2) | L CP (1)<br>L CP (2)<br>L (3) | X | R (1)<br>R (2) |
| **SUBJ 80** | Yes | L (1) | R (2)<br>R (3) | X | L (2) | **SUBJ 98** | Yes | L (1)<br>L (2)<br>L (3)<br>L (4) | R (1)<br>R (2)<br>R (3)<br>R (4) | X | L (1)<br>L (2)<br>L (4) |
| **SUBJ 81** | Yes | X | X | X | R (1)<br>R (2)<br>R (3) | **SUBJ 99** | Yes | L CP (1)<br>L (2)<br>R (3) | L CP (1)<br>R CP (2)<br>L CP (3) | X | R (1)<br>R CP (2)<br>L CP (3) |
| **SUBJ 82** | Yes | X | R (1)<br>L (3)<br>R (4) | X | X | **SUBJ 100** | Yes | L (1)<br>R (2) | R CP (1)<br>L (2)<br>L (4) | X | R CP (1)<br>R (4) |
| **SUBJ 83** | Yes | L CP (3) | R (1)<br>L (2)<br>L CP (3) | X | L (1)<br>R (2)<br>R (3) | **SUBJ 101** | Yes | R (1)<br>R (3) | L (1)<br>L (2) | X | R (2) |
| **SUBJ 84** | Yes | R (1)<br>R (2)<br>R (4) | L (1)<br>L (2)<br>L (4) | X | L(1)<br>R (3)<br>L (4) | **SUBJ 102** | Yes | L (1) | L CP (2)<br>L (3) | R CP (1)<br>L CP (2) | R CP (1)<br>R (2)<br>R (3) |
| **SUBJ 85** | No | R (4) | L (4)<br>R (3) | X | L (1)<br>L (3) | **SUBJ 103** | Yes | L (2)<br>L (3) | R (2)<br>R (3) | X | L (2) |
| **SUBJ 86** | Yes | L (1)<br>R (2)<br>L CP (4) | R CP (1)<br>L CP(2)<br>L CP (4) | X | R CP (1)<br>L CP (2)<br>R (4) | **SUBJ 104** | Yes | L CP (2) | R (1)<br>L CP (2)<br>L (3) | X | X |
| **SUBJ 87** | Yes | R (1)<br>R CP (3) | L (1)<br>L CP (3) | R CP (3) | L (2)<br>L CP (3)<br>R (4) | **SUBJ 105** | Yes | R (1)<br>R (3) | L (1)<br>L (3) | X | R (3) |
| **SUBJ 88** | Yes | R (3) | L (2)<br>L (3) | X | R (2) | **SUBJ 106** | Yes | L (1)<br>R (3) | R CP (1)<br>L CP (3) | X | R CP (1)<br>R (2)<br>L CP (3) |
| **SUBJ 107** | Yes | R CP (4) | R (2)<br>R (3)<br>R CP (4) | X | R (1)<br>L (4) | **SUBJ 123** | No | R (1)<br>L (3) | L (1)<br>L (2)<br>R (3) | X | R (1)<br>R (2)<br>L (3) |
| **SUBJ 108** | Yes | R (1)<br>L (2)<br>R (3) | L CP (1)<br>R (2)<br>L (2) | X | L CP (1) | **SUBJ 124** | No | L (4) | L (1)<br>L (3)<br>R (4) | X | R (1)<br>R (3) |

| | | | | | | | | | | | |
|---|---|---|---|---|---|---|---|---|---|---|---|
| | | | | | | | | | L (5) | | R (5) |
| **SUBJ 109** | Yes | R (1)<br>R (2)<br>R (3) | L (1)<br>L (2)<br>L (3) | X | X | **SUBJ 125** | No | R (2) | R (1)<br>L (2)<br>L (4) | X | R (3) |
| **SUBJ 110** | Yes | L (3) | L (1)<br>L (2)<br>R (3) | X | R (1)<br>R (2) | **SUBJ 126** | No | R (1)<br>L CP (3) | L (1)<br>L CP (3)<br>R (4) | L CP (3) | R (1)<br>R (2)<br>R (3)<br>L (4) |
| **SUBJ 111** | Yes | R CP (1)<br>L (2)<br>R (3) | R CP (1)<br>L (3) | X | L (1) | **SUBJ 127** | No | L CP (3) | L (1)<br>L (2)<br>L CP (3)<br>L (4) | X | R (1)<br>R (3) |
| **SUBJ 112** | Yes | R (1)<br>R(3) | L (1) | X | R (2) | **SUBJ 128** | No | L (1)<br>R (2)<br>L CP (3) | L CP (3) | X | R (1)<br>L (2)<br>R (3) |
| **SUBJ 113** | Yes | R (1)<br>R CP (2) | L CP (1)<br>R CP (2)<br>L CP (3) | L CP (3) | L CP (1)<br>L (2) | **SUBJ 129** | No | L (1)<br>L (2)<br>L (3) | R (1)<br>R (2)<br>R (3) | X | X |
| **SUBJ 114** | Yes | R (2)<br>R (3) | L (2)<br>L (3) | X | R (1) | **SUBJ 130** | No | L (1) | R (1)<br>R (2)<br>R (3) | X | L (3) |
| **SUBJ 115** | Yes | L (2)<br>R (3) | R (1)<br>R (2)<br>L (3) | X | L (1)<br>R (3) | **SUBJ 131** | No | L (2) | R (1)<br>R (2)<br>R (3) | X | L (1)<br>L (3) |
| **SUBJ 116** | Yes | L (1)<br>L (2)<br>R (3) | R (1)<br>R (2)<br>L CP (3) | L CP (3) | L (1)<br>R (3) | **SUBJ 132** | No | L (2)<br>L CP (3) | R (1)<br>R CP (2)<br>L CP (3)<br>L CP (4) | X | R CP (2)<br>R (3)<br>L CP (4) |
| **SUBJ 117** | Yes | L (1)<br>L (2) | R (1)<br>R CP (2)<br>R (3) | X | R CP (2)<br>L (3) | **SUBJ 133** | No | L CP (1) | L CP (1)<br>L (2)<br>L (4) | X | R (1)<br>R (2)<br>R (3)<br>R (4) |
| **SUBJ 118** | No | L (2)<br>L (3) | R (1)<br>R (2)<br>R (3) | X | L (1)<br>L (2)<br>L (3) | **SUBJ 134** | No | R CP (1)<br>L (2) | R CP(1)<br>R (2)<br>R (4) | X | L (1)<br>L (3)<br>L (4) |
| **SUBJ 119** | No | R (2) | R (1)<br>L (2)<br>R (3) | X | L (1)<br>L (3) | **SUBJ 135** | No | L (3) | R (1)<br>R (3)<br>R (4)<br>L (6) | X | L (1)<br>L (4)<br>L (7) |
| **SUBJ 120** | No | L (1)<br>L (2) | R (1)<br>R (2)<br>R (3) | X | L (3) | **SUBJ 136** | No | R (3) | L (2)<br>L CP (3)<br>R (4)<br>R (5) | X | L (2)<br>L CP (3)<br>L (5) |
| **SUBJ 121** | No | R (3)<br>L (4) | L (1)<br>L (3)<br>R (4) | X | R (1)<br>R (3) | **SUBJ 137** | No | LCP(5) | R (1)<br>R (3)<br>R (4)<br>L CP (5) | X | L (1)<br>L (3)<br>L (4)<br>R (5) |
| **SUBJ 122** | No | X | R (1)<br>R (2)<br>R (3) | X | L (3) | **SUBJ 138** | No | R (3) | L (2)<br>L (3) | X | R (2) |

**Supplementary Table 4. EMG and force plate recordings for stroke survivors**

*Legenda* – L: left, R; right, FP: force plate, EMG: electromyography, BWA: Basic Walk Across trials, X: no force plate data

| ID | EMG | FP 1 | FP 2 | FP 3 | FP 4 |
|---|---|---|---|---|---|
| TVC03 | Yes | L (BWA6)<br>R (BWA7) | L (BWA6)<br>R (BWA7) | X | X |
| TVC04 | Yes | R (BWA6)<br>L (BWA7) | L (BWA6)<br>R (BWA7)<br>R (BWA8) | X | R (BWA6)<br>L (BWA7)<br>L (BWA8) |
| TVC05 | Yes | L (BWA4)<br>L (BWA5) | L (BWA4)<br>L (BWA5) | R (BWA5) | L (BWA5) |
| TVC06 | Yes | L (BWA2)<br>L (BWA6) | R (BWA2)<br>R (BWA5)<br>R (BWA6) | L (BWA5) | L (BWA2) |
| TVC08 | Yes | X | X | L (BWA3) | X |
| TVC09 | Yes | R (BWA1)<br>R (BWA3) | L (BWA1)<br>R (BWA2)<br>L (BWA3) | X | R (BWA1)<br>R (BWA3) |
| TVC10 | Yes | X | X | X | X |
| TVC11 | No | X | L (BWA1)<br>L (BWA2)<br>R (BWA5) | X | X |
| TVC12 | Yes | R (BWA1)<br>L (BWA2) | L (BWA1)<br>R (BWA2)<br>L (BWA3) | X | R (BWA1)<br>R (BWA3) |
| TVC13 | Yes | L (BWA5)<br>L (BWA6) | L (BWA5)<br>L (BWA6) | R (BWA6) | L (BWA5)<br>L (BWA6) |
| TVC14 | Yes | L (BWA2)<br>L (BWA5) | L (BWA2)<br>L (BWA5) | R (BWA2)<br>R (BWA5) | L (BWA2) |
| TVC15 | Yes | R (BWA3)<br>L (BWA4)<br>R (BWA5) | L (BWA2)<br>R (BWA4)<br>L (BWA5) | X | R (BWA3)<br>L (BWA4) |
| TVC16 | Yes | X | X | X | X |
| TVC17 | Yes | X | X | L (BWA4) | X |
| TVC19 | Yes | R (BWA7) | R (BWA7) | L (BWA7) | L (BWA6)<br>R (BWA7) |
| TVC20 | Yes | L (BWA5)<br>R (BWA6) | L (BWA2)<br>R (BWA5) | X | X |
| TVC21 | Yes | R (BWA4) | X | X | R (BWA4)<br>L (BWA7) |
| TVC22 | Yes | L (BWA2)<br>R (BWA1)<br>L (BWA4) | L (BWA2)<br>R (BWA3)<br>L (BWA4) | R (BWA2)<br>L (BWA3)<br>R (BWA4) | X |
| TVC23 | Yes | X | X | X | R (BWA5)<br>L (BWA6) |
| TVC24 | Yes | X | L (BWA7) | X | L (BWA7) |
| TVC25 | Yes | R (BWA5) | R (BWA5) | L (BWA5) | R (BWA5)<br>R (BWA6) |

| | | | | | |
|---|---|---|---|---|---|
| TVC26 | Yes | R (BWA4)<br>L (BWA6) | X | X | X |
| TVC27 | Yes | L (BWA4)<br>L (BWA5) | R (BWA3)<br>R (BWA4) | X | L (BWA3)<br>L (BWA4) |
| TVC28 | Yes | X | X | X | X |
| TVC29 | Yes | X | X | X | X |
| TVC30 | Yes | R (BWA4)<br>R (BWA6) | X | X | X |
| TVC32 | Yes | R (BWA6)<br>R (BWA7)<br>R (BWA8) | L (BWA6)<br>L (BWA8) | X | R (BWA6)<br>R (BWA7)<br>R (BWA8) |
| TVC33 | Yes | R (BWA6) | R (BWA6) | L (BWA4) | R (BWA4) |
| TVC34 | Yes | X | X | X | X |
| TVC35 | Yes | L (BWA4) | X | X | L (BWA4) |
| TVC36 | Yes | X | X | L (BWA2) | X |
| TVC37 | Yes | L (BWA1) | X | X | R (BWA3) |
| TVC38 | Yes | L (BWA6) | L (BWA6) | X | X |
| TVC39 | Yes | X | X | X | X |
| TVC40 | Yes | L (BWA7)<br>R (BWA1) | R (BWA7)<br>L (BWA2) | X | R (BWA6)<br>R (BWA4) |
| TVC41 | Yes | X | X | X | R (BWA2) |
| TVC42 | Yes | X | X | X | X |
| TVC43 | Yes | L (BWA 5)<br>L (BWA 6)<br>R (BWA7) | R (BWA 5)<br>R (BWA6)<br>L (BWA7) | L (BWA6)<br>R (BWA7) | X |
| TVC46 | Yes | R (BWA6) | L (BWA5)<br>L (BWA 7) | X | X |
| TVC47 | Yes | X | L (BWA2) | X | X |
| TVC48 | Yes | X | X | X | X |
| TVC49 | Yes | L (BWA4)<br>L (BWA5) | X | X | X |
| TVC51 | Yes | L (BWA1) | L (BWA1)<br>L (BWA3) | X | X |
| TVC52 | Yes | R (BWA7) | X | X | L (BWA5) |
| TVC53 | Yes | X | L (BWA5) | X | R (BWA3) |
| TVC54 | Yes | X | X | X | X |
| TVC55 | No | R (BWA5) | X | X | R (BWA5) |
| TVC57 | No | R (BWA1) | X | X | X |
| TVC58 | Yes | X | X | X | L (BWA4) |
| TVC60 | Yes | L (BWA4)<br>R (BWA6) | R (BWA4)<br>R (BWA5)<br>L (BWA6) | X | X |

**Supplementary table 5. Walking speed (meter/second) for the able-bodied adults and stroke survivors**

Subj: subject, L: left, R: right, P: paretic, N: non-paretic

| Subj | Lside | Rside | Subj | Lside | Rside | Subj | Lside | Rside | SubJ | P side | N side |
|---|---|---|---|---|---|---|---|---|---|---|---|
| SUBJ1 | 1.0544 | 1.0673 | SUBJ55 | 1.0492 | 1.0430 | SUBJ109 | 1.2103 | 1.2258 | TVC03 | 0.2516 | 0.2543 |
| SUBJ2 | 0.9095 | 0.9064 | SUBJ56 | 1.4704 | 1.4694 | SUBJ110 | 1.1850 | 1.1951 | TVC04 | 0.9095 | 0.9034 |
| SUBJ3 | 0.8799 | 0.8798 | SUBJ57 | 1.2729 | 1.2610 | SUBJ111 | 1.3285 | 1.3359 | TVC05 | 0.1095 | 0.1136 |
| SUBJ4 | 1.0838 | 1.0883 | SUBJ58 | 1.4857 | 1.4417 | SUBJ112 | 1.1947 | 1.1654 | TVC06 | 0.6501 | 0.6530 |
| SUBJ5 | 0.8014 | 0.8042 | SUBJ59 | 1.0593 | 1.0792 | SUBJ113 | 1.1857 | 1.1941 | TVC08 | 0.1365 | 0.1405 |
| SUBJ6 | 1.3000 | 1.2975 | SUBJ60 | 1.5793 | 1.5703 | SUBJ114 | 1.1246 | 1.1305 | TVC09 | 0.8569 | 0.8661 |
| SUBJ7 | 1.1607 | 1.1611 | SUBJ61 | 1.0976 | 1.0999 | SUBJ115 | 1.0113 | 1.0213 | TVC10 | 0.3647 | 0.3634 |
| SUBJ8 | 1.1534 | 1.1599 | SUBJ62 | 1.0926 | 1.0959 | SUBJ116 | 1.0471 | 1.0665 | TVC11 | 0.4895 | 0.4872 |
| SUBJ9 | 1.0958 | 1.0977 | SUBJ63 | 1.5023 | 1.5244 | SUBJ117 | 1.2344 | 1.2343 | TVC12 | 0.6236 | 0.6121 |
| SUBJ10 | 1.0689 | 1.0692 | SUBJ64 | 1.3717 | 1.3784 | SUBJ118 | 1.0276 | 1.0284 | TVC13 | 0.2359 | 0.2342 |
| SUBJ11 | 1.1414 | 1.1333 | SUBJ65 | 1.2118 | 1.2375 | SUBJ119 | 1.1871 | 1.1830 | TVC14 | 0.2451 | 0.2418 |
| SUBJ12 | 1.1529 | 1.1274 | SUBJ66 | 1.0588 | 1.0521 | SUBJ120 | 1.2872 | 1.2724 | TVC15 | 0.8611 | 0.8705 |
| SUBJ13 | 1.0539 | 1.0516 | SUBJ67 | 1.1318 | 1.1214 | SUBJ121 | 1.2155 | 1.2325 | TVC16 | 0.3873 | 0.3820 |
| SUBJ14 | 1.1406 | 1.1771 | SUBJ68 | 1.5848 | 1.5924 | SUBJ122 | 1.1860 | 1.1524 | TVC17 | 0.6329 | 0.6059 |
| SUBJ15 | 1.0336 | 1.0355 | SUBJ69 | 1.3298 | 1.3346 | SUBJ123 | 1.0469 | 1.0388 | TVC19 | 0.4178 | 0.4132 |
| SUBJ16 | 1.1737 | 1.1647 | SUBJ70 | 1.1738 | 1.1708 | SUBJ124 | 1.0966 | 1.1040 | TVC20 | 1.3602 | 1.3325 |
| SUBJ17 | 1.1015 | 1.1231 | SUBJ71 | 1.3464 | 1.3442 | SUBJ125 | 1.3659 | 1.3592 | TVC21 | 0.9059 | 0.8903 |
| SUBJ18 | 1.1414 | 1.1222 | SUBJ72 | 1.2657 | 1.2659 | SUBJ126 | 1.4117 | 1.3981 | TVC22 | 0.3401 | 0.3402 |
| SUBJ19 | 1.0232 | 1.0165 | SUBJ73 | 1.1601 | 1.1635 | SUBJ127 | 1.2576 | 1.2728 | TVC23 | 0.2607 | 0.2563 |
| SUBJ20 | 0.9628 | 0.9566 | SUBJ74 | 1.2064 | 1.1911 | SUBJ128 | 1.7284 | 1.7609 | TVC24 | 0.1749 | 0.1789 |
| SUBJ21 | 1.0706 | 1.0692 | SUBJ75 | 1.0645 | 1.0592 | SUBJ129 | 1.0520 | 1.0177 | TVC25 | 0.3446 | 0.3510 |
| SUBJ22 | 0.9814 | 0.9755 | SUBJ76 | 1.1899 | 1.1888 | SUBJ130 | 1.2927 | 1.2581 | TVC26 | 0.4480 | 0.4467 |
| SUBJ23 | 1.0184 | 1.0051 | SUBJ77 | 1.1833 | 1.1780 | SUBJ131 | 1.1920 | 1.2107 | TVC27 | 0.6416 | 0.6532 |
| SUBJ24 | 1.1847 | 1.1753 | SUBJ78 | 1.1475 | 1.1550 | SUBJ132 | 1.7827 | 1.8032 | TVC28 | 0.3212 | 0.3464 |
| SUBJ25 | 1.1467 | 1.1373 | SUBJ79 | 1.1167 | 1.1107 | SUBJ133 | 1.1954 | 1.1809 | TVC29 | 0.1120 | 0.1132 |
| SUBJ26 | 1.2963 | 1.2944 | SUBJ80 | 1.6058 | 1.5733 | SUBJ134 | 1.3962 | 1.3998 | TVC30 | 0.3267 | 0.3345 |
| SUBJ27 | 0.8338 | 0.8148 | SUBJ81 | 1.1529 | 1.1411 | SUBJ135 | 1.1839 | 1.1821 | TVC32 | 0.7502 | 0.7614 |
| SUBJ28 | 1.0919 | 1.1002 | SUBJ82 | 1.5416 | 1.5244 | SUBJ136 | 1.3267 | 1.3413 | TVC33 | 0.2057 | 0.2093 |
| SUBJ29 | 1.2268 | 1.2117 | SUBJ83 | 1.3231 | 1.3236 | SUBJ137 | 1.3492 | 1.3575 | TVC34 | 0.5668 | 0.5657 |
| SUBJ30 | 1.4127 | 1.4188 | SUBJ84 | 1.1323 | 1.1394 | SUBJ138 | 1.1314 | 1.1385 | TVC35 | 0.4254 | 0.4391 |
| SUBJ31 | 1.3850 | 1.3964 | SUBJ85 | 1.1910 | 1.1847 | | | | TVC36 | 0.3314 | 0.3138 |
| SUBJ32 | 1.0652 | 1.0638 | SUBJ86 | 1.4056 | 1.4060 | | | | TVC37 | 0.5188 | 0.5128 |
| SUBJ33 | 1.4247 | 1.4405 | SUBJ87 | 1.2190 | 1.2071 | | | | TVC38 | 0.2248 | 0.2258 |
| SUBJ34 | 1.1887 | 1.1964 | SUBJ88 | 1.2002 | 1.1990 | | | | TVC39 | 0.2177 | 0.2235 |
| SUBJ35 | 1.3170 | 1.3316 | SUBJ89 | 1.4343 | 1.4424 | | | | TVC40 | 0.6838 | 0.6902 |
| SUBJ36 | 1.1359 | 1.1420 | SUBJ90 | 1.0378 | 1.0315 | | | | TVC41 | 0.2273 | 0.2288 |
| SUBJ37 | 1.2498 | 1.2636 | SUBJ91 | 1.3774 | 1.3507 | | | | TVC42 | 0.4274 | 0.4298 |
| SUBJ38 | 1.1878 | 1.2056 | SUBJ92 | 1.1933 | 1.1812 | | | | TVC43 | 0.8101 | 0.7997 |
| SUBJ39 | 1.0483 | 1.0454 | SUBJ93 | 1.1691 | 1.1625 | | | | TVC46 | 1.1201 | 1.1176 |
| SUBJ40 | 1.1896 | 1.1770 | SUBJ94 | 1.1644 | 1.1652 | | | | TVC47 | 0.2411 | 0.2373 |
| SUBJ41 | 1.2724 | 1.2630 | SUBJ95 | 1.2634 | 1.2757 | | | | TVC48 | 0.1958 | 0.1834 |
| SUBJ42 | 0.9759 | 0.9685 | SUBJ96 | 1.2023 | 1.2225 | | | | TVC49 | 0.2461 | 0.2602 |
| SUBJ43 | 1.4288 | 1.4206 | SUBJ97 | 1.3344 | 1.3416 | | | | TVC51 | 0.2240 | 0.2251 |
| SUBJ44 | 1.1456 | 1.1369 | SUBJ98 | 1.1551 | 1.1645 | | | | TVC52 | 1.1656 | 1.1655 |
| SUBJ45 | 1.4189 | 1.1727 | SUBJ99 | 1.3088 | 1.2992 | | | | TVC53 | 0.6975 | 0.7121 |
| SUBJ46 | 1.2879 | 1.2732 | SUBJ100 | 1.3257 | 1.3232 | | | | TVC54 | 0.3248 | 0.3245 |
| SUBJ47 | 1.1791 | 1.1666 | SUBJ101 | 1.2982 | 1.2988 | | | | TVC55 | 0.5357 | 0.5329 |
| SUBJ48 | 1.0946 | 1.1065 | SUBJ102 | 1.0499 | 1.0534 | | | | TVC57 | 0.3387 | 0.3323 |
| SUBJ49 | 1.0942 | 1.0986 | SUBJ103 | 1.0001 | 1.0064 | | | | TVC58 | 0.7926 | 0.8038 |
| SUBJ50 | 0.9895 | 1.0005 | SUBJ104 | 1.4896 | 1.4750 | | | | TVC60 | 0.8258 | 0.8251 |
| SUBJ51 | 1.4176 | 1.4258 | SUBJ105 | 0.9778 | 0.9783 | | | | | | |
| SUBJ52 | 1.0400 | 1.0342 | SUBJ106 | 1.2414 | 1.2354 | | | | | | |
| SUBJ53 | 1.5175 | 1.5686 | SUBJ107 | 1.2305 | 1.2366 | | | | | | |
| SUBJ54 | 1.4960 | 1.4728 | SUBJ108 | 1.3310 | 1.3506 | | | | | | |
